# Supplementary material for: MetaRibo-Seq measures translation in microbiomes
Source: Nat Commun. 2020 Jun 29;11:3268. doi: 10.1038/s41467-020-17081-z (PMC7324362; doi:10.1038/s41467-020-17081-z)
Supplement: Supplementary file 10 — Supplementary Data 7 [file 41467_2020_17081_MOESM10_ESM.zip › File2/Confidence_High_Taxonomy/235230_out.krona.html]

Javascript must be enabled to view this page.

members
magnitude
magnitudeUnassigned
count
unassigned
taxon
rank

235230\_out

3

2
superkingdom
3

1239
3
phylum

class
3
186801

186802
order
3

31979
family
1

1485
1
genus

2293014
1

SRS149181\_contig\_number\_28775
species

family
1
186806

genus
1
1730

39485
species

SRS893270\_contig\_number\_22656
1

186803
family
1

572511
1
genus

species

SRS104975\_contig\_number\_7829
1
2292959
